# The Prognostic and Immunotherapeutic Significance of AHSA1 in Pan-Cancer, and Its Relationship With the Proliferation and Metastasis of Hepatocellular Carcinoma

**DOI:** 10.3389/fimmu.2022.845585

**Published:** 2022-06-10

**Authors:** Wenli Li, Jun Liu

**Affiliations:** ^1^Reproductive Medicine Center, Yue Bei People’s Hospital, Shantou University Medical College, Shaoguan, China; ^2^Medical Research Center, Yue Bei People’s Hospital, Shantou University Medical College, Shaoguan, China

**Keywords:** AHSA1, HCC, immune, cell migration, prognosis

## Abstract

The *AHSA1* is a main activator of ATPase of Hsp90. Hsp90 is involved in various metabolic and developmental processes of tumor cells. Although, the role of *AHSA1* in tumor cells is still unrecognized. In the current research, the RNA-seq of 33 tumors were downloaded using The Cancer Genome Atlas (TCGA) database for the analysis of *AHSA1* expression in tumors. The Kaplan-Meier method was used for the evaluation of the prognostic significance of *AHSA1* in patients with pan-cancer. Additionally, the correlation between *AHSA1* and immune cell infiltration, immune checkpoint, pyroptosis-related molecules, epithelial cell transformation-related molecules, and autophagy-related molecules were analyzed by co-expression. Furthermore, we examined the effect of *AHSA1* knockdown on cell function in Huh7 and HCCLM3 cells of hepatocellular carcinoma (HCC) cell lines.

According to the finding of this study, up-regulation of *AHSA1* expression was observed in numerous tumor tissues, and its over-expression in liver hepatocellular carcinoma (LIHC), lung adenocarcinoma (LUAD), and esophageal carcinoma (ESCA) could affect the overall survival and disease-specific survival of the patients. Meanwhile, as per the correlation analysis the expression of *AHSA1* was greatly correlated with the expression of various immune cell infiltrates, immune checkpoint inhibitors, tumor mutation load, and microsatellite instability. Moreover, this study focused on analyzing the association of *AHSA1* expression with multiple pathological stages in HCC, and confirmed that *AHSA1* was an independent prognostic factor of HCC by univariate and multivariate COX regression in TCGA and The International Cancer Genome Consortium (ICGC) cohorts. At the same time, cellular experiments proved that the *AHSA1* knockdown could decrease the proliferation activity, cell migration and invasion ability of HCC cells. Therefore, the results of this study indicated that *AHSA1* can be used as a potential prognostic biomarker of tumors and it may have a significant role in the proliferation as well as migration of HCC cells.

## Introduction

In recent decades, the malignant tumor has shown an alarming incidence and mortality rate worldwide, and it is a major threat to people’s health in the 21st century (1). Although the increase of mortality rate of cancer patients in developed countries has moderated its steps in recent years, thanks to advanced prevention and treatment concepts and medical technologies, the mortality rate of cancer patients around the world still shows a prominent growth trend (2). With research development, a growing number of researchers began to pay attention to the common characteristics of various human malignant tumors, to explore the potential mechanism of tumor genesis (3). At present, pan-cancer research has been widely used to identify tumor molecular markers and signal pathways, combined with multiple omics analysis, in order to understand more comprehensively and deeply the molecular mechanism of tumor genesis and development (4–6). As a co-molecular chaperone protein, AHSA1 is currently the only ATPase activator of Hsp90 (7). AHSA1 plays a key role in regulating the molecular chaperone cycle and protein folding of Hsp90 (8). Hsp90, the acting protein of AHSA1, is a classical molecular chaperone with a highly conserved molecular structure. It can facilitate protein folding, maturation and transport of some cancer-related proteins, such as BCR-ABL, ErbB2/Neu, Akt, HIF-1α, p53 and RAF-1 (9). Lindsey B. Sheltont et al. revealed that the inhibition of *AHSA1* expression in mice models of Alzheimer’s disease and *in vitro* cell models can eliminate the accumulation of Tau protein caused by Hsp90, which is expected to become a new therapeutic target for Alzheimer’s disease (10). According to Jianli Shao et al, *AHSA1* mediates the invasion, proliferation, and apoptosis of tumor cells by regulating the Wnt/β-catenin pathway in osteosarcoma. At the same time, after the knockdown of AHSA1, the ATPase activity of Hsp90 decreased (11). However, current studies are limited to a few types of cancers, and the role of *AHSA1* in various types of tumors is still not understood completely. In this study, the RNA-seq from TCGA was utilized for the examination of *AHSA1* expression in a variety of human tumors. Combined with clinical follow-up data, the impact of abnormal expression of *AHSA1* on the prognosis of cancer patients was analyzed. Meanwhile, combined with correlation analysis, the possible association between *AHSA1* expression and tumor immune cell infiltration, tumor mutation load (TMB), microsatellite instability (MSI) and various pathway-related molecules were explored. In addition, this study verified the expression and prognostic role of *AHSA1* in hepatocellular carcinoma by combining data from ICGC-LIRI-JP, and verified the expression and potential function of *AHSA1* in hepatocellular carcinoma by combining cell and tissue specimens. The results of this study are helpful to understand the similarities and differences of *AHSA1* expression in a variety of tumors, reveal the potential mechanism of the interaction between *AHSA1* and tumor immunity, and demonstrate the potential function of *AHSA1* in hepatocellular carcinoma.

## Materials and Methods

### Data Collection of Patients’ Data Sets

The count format of 33 common tumor transcriptome data was downloaded from TCGA’s official website and converted into the TPM format. The number of tumors of each type was shown on the **Table S1**. The clinical follow-up survival and staging data were downloaded at the same time. The transcriptome data of hepatocellular carcinoma was provided by the ICGC database and converted into the TPM format, and corresponding clinical data were obtained. In addition, the single-nucleotide mutation data of 33 tumors were downloaded from TCGA’s official website and their tumor mutation load was calculated.

### Differential Analysis and Prognostic Analysis

Transcriptome data from 33 tumors were filtered by “RMA” packs to remove the NA and duplicates, and log2 (TPM +1) conversion was performed afterward. Then, for comparing the expression differences of *AHSA1* in the normal and tumor tissues in different tumors, the Wilcoxon rank sum test was performed. Using the “SurvMiner” and “Survival” packages, the median expression values based on *AHSA1* were classified into two groups; the high risk and low-risk groups in different kinds of tumors. Their survival curves were drawn using the Kaplan-Meier method, and their statistical significance was calculated by the log-rank test.

### Correlation Analysis

A comprehensive website; Tumor Immune Estimation Resource (TIMER, https://cistrome.shinyapps.io/timer/) was used for the measurement of the level of tumor immune cells infiltrating degree (12). For predicting the immune cell infiltration level from tumor transcription data there are five different methods provided by TIMER website which includes TIMER, CIBERSORT, xCell, McP-counter and EPIC. The abundance of infiltrating immune cells in tumor samples from 33 tumor types was obtained from TIMER database. The single-sample gene set enrichment analysis (ssGSEA) was performed in R package GSVA. Subsequently, the relation of *AHSA1* expression and the level of infiltration of these immune cells was calculated. At present, TMB and MSI are considered for use as potential biomarkers to predict the efficacy of tumor immunotherapy (13, 14). The simple nucleoside variation data of level 4 processed with MuTect2 software was acquired from the TCGA database, and the TMB was calculated (15). Microsatellite instability signature for 33 tumors were obtained from reported studies (16). Reports have indicated that Epithelial-Mesenchymal Transitions (EMT), Pyroptosis and Autophagy were all associated with tumor metastasis and malignant proliferation, and the correlation between *AHSA1* and the expression of these pathway molecules was also analyzed in this study.

### Protein Network Construction and Gene Enrichment Analysis

The GeneMANIA (http://genemania.org/) database finds out functionally identical genes on the basis of genomic and proteomic data (17). The GeneMANIA database was used to predict the genes that have functions similar to that of *AHSA1*. Gene oncology (GO) and Kyoto Encyclopedia of Genes and Genomes (KEGG) analyses of molecules with potential roles in AHSA1 were performed using clusterProfiler packages.

### Correlation Analysis of *AHSA1* Expression and Clinical Factors

We analyzed the correlation between *AHSA1* expression and clinical stage, including pathologic stage, tumor (T) stage, histologic grade, alpha-fetoprotein (AFP) level, and vascular invasion. Meanwhile, for the further evaluation of the prognostic value of *AHSA1* in HCC patients, univariate and multivariate COX regression and Receiver Operating characteristic curve (ROC) analysis were performed respectively. In addition, we constructed a nomography based on the expression value of *AHSA1* and pathologic stage to promote the application of *AHSA1* in the evaluation of clinical prognosis of hepatocellular carcinoma, and evaluated the prediction accuracy of the nomography by calibration curve.

### Cell Culture

The American Type Culture Collection (ATCC, Manassas, VA, United States) provided the normal human liver cells LO2, HCC cell lines HepG2, Huh7, and HCCLM3 for this experiment. All cells were cultured in an incubator at 37 °C with 5% CO2. Human target gene AHSA1 short hairpin RNA (shRNA) was purchased from GenScript company (https://www.genscript.com/), the sequences are as follows:

*AHSA1* shRNA-1:gGGTGAAACTTCTAAGAGAAttcaagagaTTCTCTTAGAAGTTTCACCttttt, *AHSA1* shRNA-2:gGGCATGATCTTACCTACAAttcaagagaTTGTAGGTAAGATCATGCCttttt,

*AHSA1* shRNA-3:gAGTCAGGAGTACAATACAAttcaagagaTTGTATTGTACTCCTGACTttttt. Huh7 and HCCLM3 cells were plated in six-well plates (4 × 10^5^ cells/well), and cultivated in a in a 37°C, 5% CO2 incubator until they were completely adherent to the wall. Subsequently, the cells were transfected with lipo3000.

### Quantitative Reverse Transcription-Polymerase Chain Reaction, RT-PCR

Four types of cell strains were used for total RNA was extraction. Reverse transcription was performed to convert the extracted RNA into cDNA using the reverse transcription kit provided by Beyotime, https://www.beyotime.com/. Exicycler 96 (BIONEER) was used to detect its fluorescence expression quantity. All results were processed with *GAPDH* for standardization. The 2^-ΔΔ Ct^ method was used to calculate the relative expression levels of genes.

### Immunohistochemistry and Western Blot Analysis

Ten pairs of paraffin sections of hepatocellular carcinoma tumor tissue specimens and para-cancer specimens were provided by the Department of Pathology, North Guangdong People’s Hospital. AHSA1 antibody was purchased from Proteintech Company (Article number: 14725-1-AP). Approval for this research was given by the Ethics Committee of North Guangdong People’s Hospital. Western blotting was performed for the detection of post knockout changes in AHSA1 protein levels.

### CCK8 Detection

HuH7 cells transfected with shRNA-*AHSA1* were digested when their level reached 90%, and then they were inoculated into 96-well culture plates with 3×10^3^cells per well, and 5 multiple wells were designed for each group. Then, they were cultured in a 37°C, 5% CO2 incubator, and tested at 0h, 24h, 48h, 72h and 96h using the CCK-8 kit (WLA074, China).

### Wound Healing Test

6-well plates were used for the inoculation of these cells, and after 48 hours of transfection, a 200 μl pipette tip was used to scratch the cells. The cell surface was cleaned with serum-free medium once, and the cell fragments were removed. The cells were then observed and photographed under a 100× microscope, and their positions in the photos were recorded. Subsequently, cells in each group were placed in an incubator at 37 °C with 5% CO2 for 24h and 48h, and then photographed and recorded, and the mobility of each group was calculated.

### Transwell Migration and Invasion Assay

A 24-well Transwell chamber (8 μm aperture; Corning Costar, USA) was prepared overnight at 4°C with or without 100μL matrix gel substrate provided by BD Biosciences, San Jose, CA, USA. A 200ul of cell suspension containing 1×10^5^cells/mL was inoculated into Transwell cells with or without matrix glue, and a culture medium (800 ul) containing 10% FBS was poured into the lower chamber. After 24h culture, cell fixation was done using 4% paraformaldehyde at room temperature for 20 minutes and staining was performed for 5 minutes with 0.5% crystal violet dye. The cell count was recorded afterwards.

## Results

### The *AHSA1* Expression in Pan-Cancer

**Figure 1:**
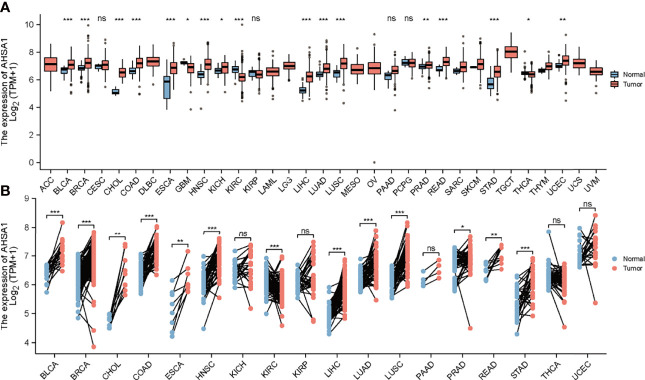
Differential expression analysis of *AHSA1*. **(A)** Expression of *AHSA1* mRNA in pan-cancer. **(B)** The expression differences of *AHSA1* in tumor and corresponding adjacent tissues were compared with paired analysis. Mann-Whitney U test was used for this analysis, ns, p≥0.05; *p< 0.05; **p<0.01; ***p<0.001.

The mRNA expression of *AHSA1* was evaluated in pan-cancer patients according to the RNA-seq data of 33 types of TCGA tumors. The results showed that *AHSA1* was expressed at a relatively low level in normal bile duct tissue and a relatively high level in testicular germ cell tumor tissue. At the same time, the variance analysis indicated a relatively high expression of *AHSA1* in Bladder Urothelial Carcinoma (BLCA), Breast invasive carcinoma (BRCA), Cholangiocarcinoma (CHOL), Colon adenocarcinoma (COAD), Esophageal carcinoma (ESCA), Head and Neck squamous cell carcinoma (HNSC), kidney chromophobe (KICH), LIHC, Lung adenocarcinoma (LUAD), Lung squamous cell carcinoma (LUSC), Rectum adenocarcinoma (READ) and stomach adenocarcinoma (STAD), Prostate adenocarcinoma (PRAD), and uterine corpus endometrial carcinoma (UCEC) tumor tissues as compared to the corresponding para-carcinoma tissue (**Figure 1A**). Furthermore, paired comparison analysis was performed and the results of this analysis also revealed the overexpression of *AHSA1* in BLCA, BRCA, CHOL, COAD, ESCA, HNSC, LIHC, LUAD, LUSC, PRAD, READ, STAD in tumor tissues (**Figure 1B**). Considering that AHSA1 is an ATPase activator of HSP90AA1, we analyzed the expression of *HSP90AA1* in pan-cancer. Interestingly, the similar results were observed for *HSP90AA1* expression in pan-cancer (**Figures S1A, B**).

### Survival Analysis

**Figure 2:**
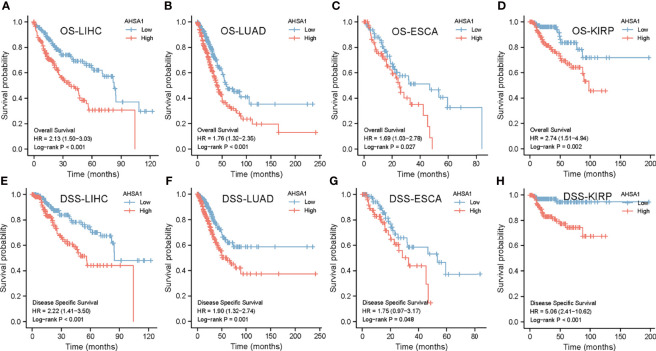
Survival analysis of *AHSA1* in pan-cancer. The effect of *AHSA1* expression on the overall survival rate (OS) of LIHC **(A)**, LUAD **(B)**, ESCA **(C)** and KIRP **(D)** was analyzed by Kaplan-Meier method. Meanwhile, the influence of abnormal expression of *AHSA1* on disease-specific survival (DSS) of LIHC **(E)**, LUAD **(F)**, ESCA **(G)** and KIRP **(H)** was calculated.

Based on the TCGA database, the Kaplan-Meier curves helped in depicting the association between abnormal expression of *AHSA1* and the general pan-cancer survival. The results indicated that the survival rate of patients with high expression levels of *AHSA1* in LIHC, LUAD, ESCA, and KIRP was worse (**Figures 2A–D**). For further evaluation of the specific effect of AHSA1 on tumor survival, the influence of abnormal *AHSA1* expression on disease-specific survival (DSS) of tumor patients was observed. The Kaplan-Meier plots showed that the abnormally increased *AHSA1* in LIHC, LUAD, ESCA and KIRP was correlated with its poor DSS (**Figures 2E–H**).

### Expression of *AHSA1* and Analysis of Tumor Microenvironment

**Figure 3:**
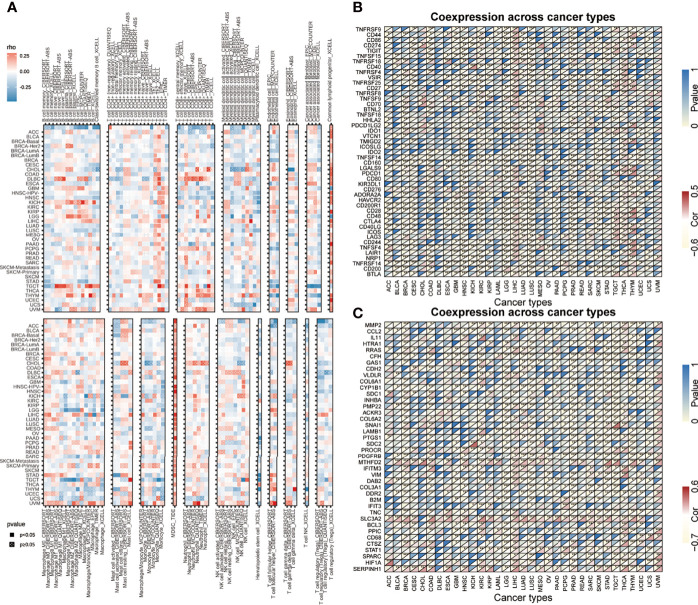
Correlation analysis between the expression of *AHSA1* and immune cell infiltration, checkpoint inhibitors and pyroptosis-related molecules. **(A)** Correlation analysis between *AHSA1* and immune cell infiltration in pan-cancer. **(B)** Correlation analysis between the expression of *AHSA1* and immune checkpoint inhibitors. **(C)** Coexpression analysis of *AHSA1* expression and epithelial-mesenchymal transition-related molecules. *p< 0.05; **p<0.01; ***p<0.001.

**Figure 4:**
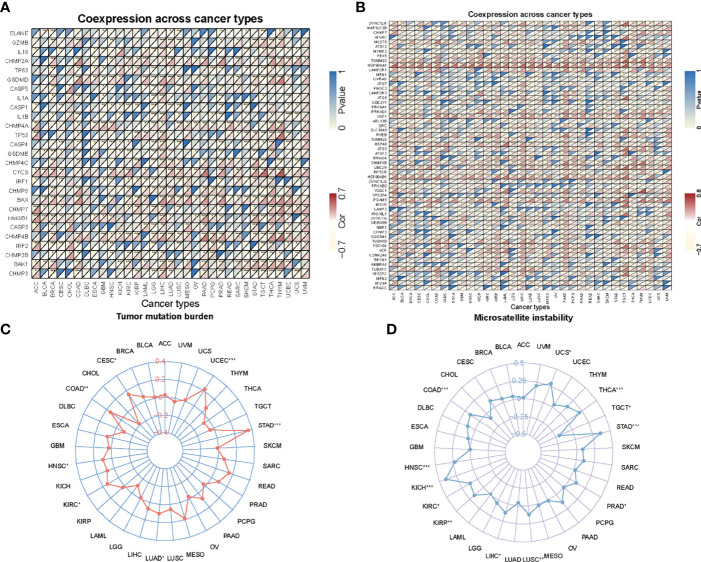
Correlation analysis. **(A)** Correlation analysis of *AHSA1* expression and pyroptosis-related molecules. **(B)** Correlation analysis of *AHSA1* expression and autophagy-related molecules. **(C)** Correlation between *AHSA1* expression and Tumor mutation burden. **(D)** Correlation analysis of *AHSA1* expression and Microsatellite instability. *p<0.05, **p<0.01, ***p<0.001.

Tumor microenvironment is a complicated survival environment of tumor cells, mostly comprising of immune cells, mesenchymal environment as well as related internal and external molecular composition (18). In recent years, growing evidence indicate that the immune cell infiltration level is closely associated with the survival of tumor cells. Although previous studies have suggested the prognostic importance and expression level of *AHSA1* in various types of cancers, little is known if the abnormal expression of *AHSA1* have an influence on immune cell infiltration. In this study, the correlation between abnormal *AHSA1* expression and immune cell infiltration was evaluated using a TIMER database based on multiple immune prediction methods. The results indicated that *AHSA1* was substantially positively correlated with CD4+Th1, CD4+Th2 and MDSC cells of various tumors (**Figure 3A**). Meanwhile, *AHSA1* showed a significant positive correlation with B cells, CD4+ T cells, CD8+T cells and Myeloid dendritic cells in LIHC (**Figure 3A**). Moreover, we discussed the correlation between the *AHSA1* expression and immune checkpoint inhibitor. The results showed that *AHSA1* was significantly positively correlated with several immune checkpoint inhibitors, especially in LIHC, THCA, and THYM. Meanwhile, *AHSA1* was significantly negatively correlated with several immune checkpoint in ACC, BRCA, GBM, HNSC, LUSC, LGG, and PRAD (**Figure 3B**). In addition, *AHSA1* was significantly positively associated with *CD274, CD276*, and *CTLA4* in LIHC, THCA, and TGCT (**Figure 3B**). It is understood that EMT has a significant function in tumor metastasis, which is the major cause of death. To investigate whether *AHSA1* is related to tumor metastasis, the link between *AHSA1* expression and the expression of EMT-related molecules was analyzed. Results show a significant positive correlation between the *AHSA1* and EMT-related molecules in LIHC, KIRC, and UVM (**Figure 3C**). Additionally, the *AHSA1* and *MTHFD2, SLC3A2* and *SERPINH1* showed a significant positive correlation in a wide variety of tumors. The *AHSA1* and *MMP2, CCL2, CFH*, and *CYP1B1* showed a significant negative correlation in a variety of tumors (**Figure 3C**). Pyroptosis is a new type of programmed inflammatory cell death that has a significant function in the development of tumor. Hence, the expression correlation between *AHSA1* and pyroptosis-related molecules was observed in this study. The results showed that *AHSA1* showed a significant positive correlation with pyroptosis-related molecules in BRCA, LGG, LIHC, PRAD, and THCA (**Figure 4A**). In addition, we also discussed the *AHSA1* correlation with autophagy-related molecules, the results also showed a significant positive correlation between *AHSA1* and autophagy-related molecules in BRCA, COAD, LIHC, LUAD, PAAD, PRAD and TGCT. Similar to the previous trends, *AHSA1* was significantly positively correlated with *HSP90AA1* and *HSP90AB1* (**Figure 4B**). In addition, tumor mutation load (TMB) and microsatellite instability (MSI) are known as important factors that affect tumor genesis and development. Therefore, the association between TMB or MSI and *AHSA1* expression was observed in 33 commonly known types of cancers. Results show that the *AHSA1* presented substantial relation with TMB in CESC, COAD, HNSC, KIRC, LUAD, STAD, and UCS (**Figures 4C, D**).

### Molecular Interaction Network and Enrichment Analysis

**Figure 5:**
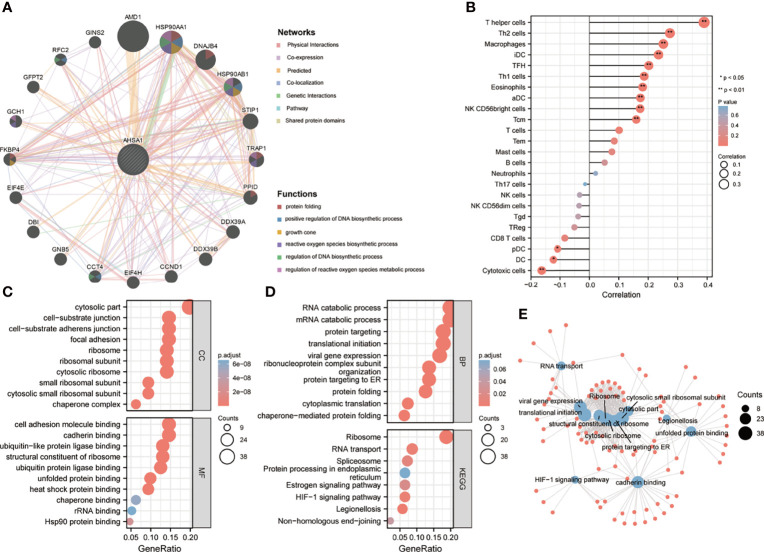
Potential functions of *AHSA1*. **(A)** The potential interaction molecular network of *AHSA1* was created using the GeneMANIA. **(B)** The ssGSEA algorithm was employed to evaluate the abundance of immune cell infiltration in HCC. GO and KEGG functional enrichment analysis of the molecules interacted with AHSA1 **(C, D)**. **(E)** Construction of GO and KEGG interaction networks.

To deeply understand the possible role of *AHSA1*, the molecules interacting with *AHSA1* were analyzed by GeneMANIA and comPPI. The results from GeneMANIA showed that *AHSA1* had potential interactions with *AMD1, HSP90AA1, DNAJB4* and *HSP90AB1*. Main functions included protein folding, up-regulation of DNA biosynthetic process and regulation of the metabolic processes of reactive oxygen species (**Figure 5A**). Single-sample gene set enrichment analysis (ssGSEA) algorithm was introduced to predict the infiltration levels of 24 immune cell types in HCC. The results indicated that *AHSA1* was positively correlated with T helper cells, Th2 cells, Macrophages, and iDC (**Figure 5B**). While, cytotoxic cells and Dendritic cells was negatively correlated with AHSA1 expression in HCC (**Figure 5B**). To further identify the possible role of *AHSA1*, the GO and KEGG enrichment analysis was performed on molecules that interacted with AHSA1 obtained from GeneMANIA. Cellular Component enrichment analysis showed that these molecules are mainly enriched in the cytosolic part, cell-substrate junction, cytosolic ribosome, and cytosolic small ribosomal subunit. The analysis of molecular function enrichment indicated that these molecules mostly play a role in cell adhesion molecule binding, cadherin binding, ubiquitin-protein ligase binding, stressed protein binding, heat shock protein binding, and Hsp90 protein binding (**Figure 5C**). Enrichment analysis of biological processes showed that these molecules are mainly involved in RNA catabolic process, mRNA catabolic process, protein targeting, protein folding, and cytoplasmic translation (**Figure 5D**). KEGG analysis showed that these molecules were mainly involved in ribosome, RNA transport, Spliceosome, *HIF-1α* signaling pathway and Legionellosis (**Figure 5D**). In addition, we built an interactive relationship network between GO and KEGG, as shown in **Figure 5E**.

### Clinical Correlation Analysis of AHSA1 in Hepatocellular Carcinoma

**Figure 6:**
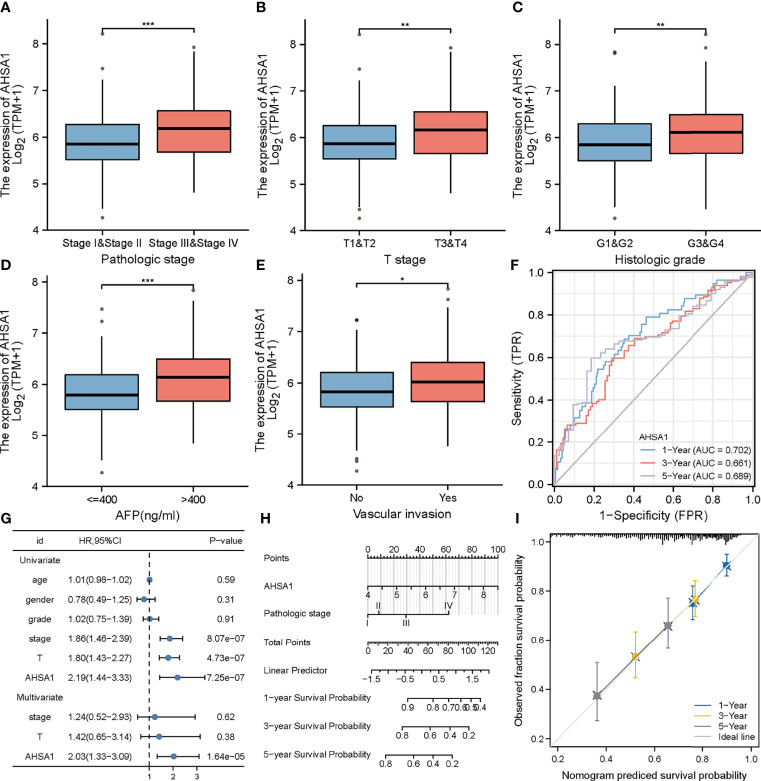
Clinical correlation analysis of *AHSA1* in hepatocellular carcinoma. Variation analysis of *AHSA1* expression in different pathological stages **(A)**, T stage**(B)**, Histologic grade **(C)**, alpha-fetoprotein (AFP) **(D)**, Vascular invasion **(E)**. **(F)** Prognostic significance of *AHSA1* in hepatocellular carcinoma was analyzed by COX analysis. **(G)** Prognostic significance of *AHSA1* in hepatocellular carcinoma was analyzed by univariate and multivariate COX. **(H)** Nomogram based on *AHSA1* expression and pathological staging. **(I)** Correction analysis diagram of the nomogram. *p<0.05, **p<0.01, ***p<0.001.

**Figure 7:**
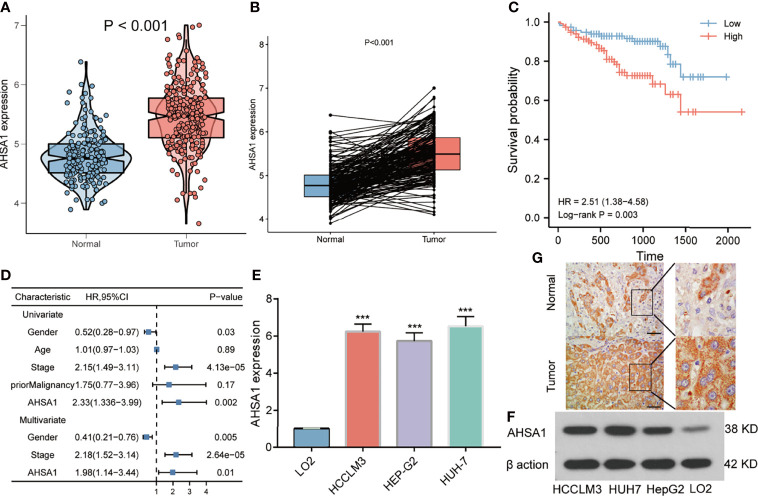
Validation of *AHSA1* expression in hepatocellular carcinoma. **(A)** The mRNA expression of *AHSA1* in ICGC cohort. **(B)** Comparing the *AHSA1* expression in paired normal and tumor. **(C)** The relationship between *AHSA1* expression and overall survival in ICGC cohort. **(D)** Overexpression of *AHSA1* was an independent prognostic marker of HCC patients in ICGC cohort. **(E)** Relative mRNA expression of *AHSA1* in HCC cell lines. **(F)** Expression levels of AHSA1 protein in HCC cell lines. **(G)** Representative immunohistochemical analysis of *AHSA1* in HCC. ***p<0.001.

The findings of this study suggests that *AHSA1* may perform a key role in the development of hepatocellular carcinoma. Subsequently, the association between *AHSA1* expression and clinical stage was analyzed in hepatocellular carcinoma. The results indicated that *AHSA1* had a higher expression level in the higher pathologic stage (**Figure 6A**), T stage (**Figure 6B**), and histologic grade (**Figure 6C**). Meanwhile, *AHSA1* expression was also related to the expression level of AFP and vascular invasion status (**Figures 6D, E**). These findings indicate that *AHSA1* expression may be linked to the malignant pathological progression of hepatocellular carcinoma. Subsequently, we analyzed the prognostic evaluation efficacy of *AHSA1* in the overall survival rate of HCC by ROC, and the results showed that *AHSA1* showed good prognostic evaluation performance in HCC, with AUC of 0.702, 0.661 and 0.689 at 1, 3 and 5 years respectively (**Figure 6F**). We further evaluated the prognostic role of *AHSA1* in hepatocellular carcinoma by univariate and multivariate COX regression combined with clinical data, and the results showed that *AHSA1* was an independent prognostic factor of hepatocellular carcinoma (**Figure 6G**). Furthermore, in order to promote the application of *AHSA1* in clinical evaluation, we constructed a nomography (**Figure 6H**) based on the expression of *AHSA1* and the pathologic stage. At the same time, we evaluated the accuracy of the model for prognosis assessment of hepatocellular carcinoma patients after 1, 3 and 5 years by calibration curve, and according to the results, the nomography had very good accuracy, almost close to the ideal model (**Figure 6I**). For further confirmation of the value of prognosis of *AHSA1* in HCC, the expression of *AHSA1* in para-cancer and tumor tissues in the ICGC-LIRI-JP queue were analyzed. Both unpaired analysis (**Figure 7A**) and paired analysis (**Figure 7B**) showed that *AHSA1* has higher expression in HCC tumor tissues. Meanwhile, K-M curve analysis showed that HCC patients with high *AHSA1* expression in the ICGC queue had poorer survival expectations (**Figure 7C**). Meanwhile, the univariate and multi-variate COX analyses showed that *AHSA1* was an independent prognostic risk factor for HCC (**Figure 7D**). For additional verification of the expression of *AHSA1* in hepatocellular carcinoma, RT-PCR was performed to detect the mRNA expression levels of *AHSA1* in normal hepatocyte cell line LO2 along with three hepatocellular carcinoma cell lines, HCCLM3, HepG2 and Huh7. Results showed higher mRNA expression levels of *AHSA1* in hepatocellular carcinoma cells (**Figure 7E**). We also confirmed that the AHSA1 protein is expressed at a higher level in HCC cells compared to normal cells via western blot (**Figure 7F**). Subsequently, the protein expression level of AHSA1 were observed in hepatocellular tissues by immunohistochemistry, and according to the findings of this analysis, the level of protein expression of AHSA1 was greater in hepatocellular carcinoma tissues (**Figure 7G**).

### The Effects of *AHSA1* on Proliferation, Migration, and Invasion of Hepatocellular Carcinoma Cells

**Figure 8:**
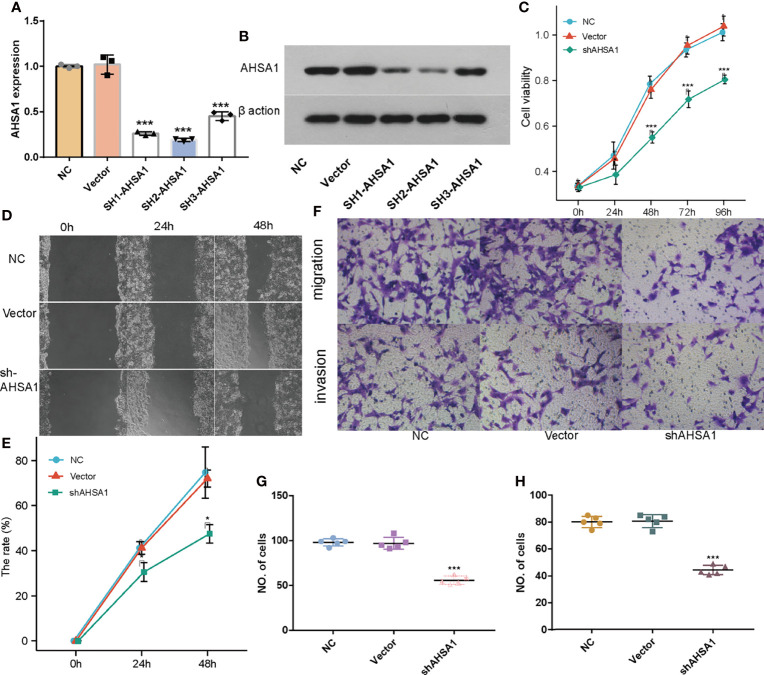
Effects of *AHSA1* knockdown on Huh7 cell proliferation and migration. Real time PCR **(A)** and Weston blot **(B)** determine the efficiency of *AHSA1* knockdown in Huh7 cells. **(C)** Huh7 cell proliferation was detected by CCK8 assays. **(D, E)**. Cell migration ability was examined by cell scratch assay. **(F)** Tranwell analysis was preformed to determine the cell migration **(G)** and invasion **(H)**. *p<0.05, ***p<0.001.

**Figure 9:**
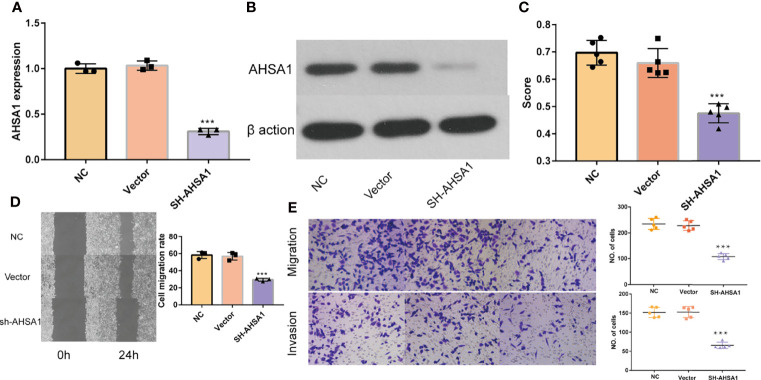
Effects of *AHSA1* knockdown on HCCLM3 cell proliferation and migration. Real time PCR **(A)** and Weston blot **(B)** determine the efficiency of *AHSA1* knockdown in Huh7 cells. **(C)** CCK8 assays was performed to detect the cell proliferation of HCCLM3. **(D)** Scratch test was used to determine the cell migration. **(E)** Cell migration and invasion was detected by Tranwell assay. ***p<0.001.

Although the previous findings indicated that *AHSA1* was significantly over-expressed in hepatocellular carcinoma and patients with overexpression had poorer survival expectations, the role of *AHSA1* in the occurrence of hepatocellular carcinoma needs further study. Firstly, three shRNA knockout carriers of *AHSA1* were constructed and transfected into HUH7 cells. The RT-PCR and Western blot showed variations in mRNA and protein levels, respectively. According to the results, the SH2-*AHSA1* had the highest knockout efficiency (**Figures 8A, B**), and we named the carrier as sh*AHSA1*. Subsequently, we tested the changes of cell activity at different time points after transfection in three groups, namely normal cell group, negative vector group and sh*AHSA1* transfection group, using the CCK8 kit. The results showed that 48 hours after *AHSA1* knockdown, the proliferation ability of cells was significantly reduced (**Figure 8C**). In addition, after cell scratch test, we found that the healing ability of *AHSA1* knockout cells was significantly weakened (**Figures 8D, E**). Furthermore, the Transwell chamber experiment was performed to verify that the migration ability (**Figures 8F, G**) and invasion ability (**Figures 8F, H**) of Huh7 cells were significantly weakened after *AHSA1* knockout. In additional, the knockdown efficiency of *AHSA1* in HCCLM3 cells was validated by RT-PCR and Western blot (**Figures 9A, B**). CCK8 assays showed that knockdown of *AHSA1* significantly inhibited cell proliferation after 24 hours transfection in HCCLM3 cells (**Figure 9C**). Furthermore, scratch assays and transwell experiment showed that knockdown of *AHSA1* markedly reduced migration/invasion ability of HCCLM3 cell (**Figures 9D, E**). There was significant positive correlation between the mRNA expression level of *AHSA1* and *Hsp90AA1.* In order to further explore the relationship between *AHSA1* and *Hsp90AA1*, we knocked down *AHSA1* in Huh7 cells and examined the effect on *Hsp90AA1*. The rt-PCR and Western blot analysis showed that the knockdown of *AHSA1* significantly reduced the *Hsp90AA1* mRNA and protein levels (**Figures S2A, B**).

## Discussion

Heat Shock protein 90 (Hsp90) is a highly conserved, extensively exit molecular chaperone that has a role in the stabilizing and proper folding of at least 300 proteins (19). Reportedly, the Hsp90 protein is known to have a significant function in various cellular processes, signaling, tumor metastasis and tumor immunity (20). Hsp90 is a kind of molecular chaperone highly dependent on ATP activity, and its executive function also requires the cooperation of various auxiliary chaperone molecules. *AHSA1* can strongly stimulate the ATPase of Hsp90 and plays a significant role in the executive function of Hsp90 (21). Moreover, *AHSA1* itself is also a helper molecular chaperone involved in the maturation and stabilization of various proteins. However, systematic studies on the expression and role of *AHSA1* in tumors are still inefficient.

According to this study, based on the TCGA database, *AHSA1* was abnormally overexpressed in different types of tumors through unpaired and paired comparisons, but was expressed in low quantities in the KIRC tumor tissues. Meanwhile, by using the K-M survival analysis method, it was found that abnormally high expression of *AHSA1* in LIHC, LUAD, ESCA, and KIRP was associated with poor OS and DSS. These findings indicate that *AHSA1* may have a significant role in tumor genesis and prognosis. The tumor immune microenvironment can interact with tumor cells and play a significant function in tumor cell clearance and immune escape (22). Several reports have suggested that immune cell infiltration level in the tumor immune microenvironment is associated to tumor development and prognosis (23, 24). This study reported that the expression of *AHSA1* was significantly positively related to the infiltration level of CD4+ T cells and MDSC cells in a variety of tumors. Moreover, ssGSEA analysis also confirmed that *AHSA1* was substantially positively associated with infiltration levels of T helper cells, Th2 cells and Macrophages cells in LIHC. B Th2 cells are known to inhibit Th1 cells differentiation and IFN-γ-secreting but promote tumor cell proliferation *via* secretion of IL-4 and IL-10 (25, 26). As an important part of tumor immune microenvironment, macrophages have attracted more and more attention (27, 28). M2 macrophage can be induced to differentiate by IL-4, and promote tumorigenesis and tumor progression (29). Therefore, we preliminarily speculated that *AHSA1* might promote the tumor progression through regulating the polarization of Th2 and macrophages cells. Immune checkpoint is an important immunomodulator to maintain immune homeostasis and prevent autoimmunity (30). It consists of stimulant and inhibitory pathways, which are important for maintaining autoimmune tolerance and regulating the type, intensity, and duration of immune responses (31). The results of this study showed that *AHSA1* was substantially linked with immune checkpoints in BRCA, LIHC, and LUSC. According to these findings the *AHSA1* may play a certain role in the regulation of tumor microenvironment. Epithelial mesenchymal cell transformation, pyroptosis, and autophagy are significantly involved in the genesis, progression, and metastasis of tumors (32–34). The results of this study also indicate that *AHSA1* expression is significantly positively co-expressed with these pathway-related molecules in a variety of tumors. These results suggest that abnormal expression of *AHSA1* may be associated with tumor genesis and metastasis. Moreover, molecules with possible effects of *AHSA1* were also explored and the molecular interaction network was constructed. As expected, *AHSA1* showed strong interaction with *AMD1* and *HSP90*, which is consistent with previous reports (35). At the same time, by conducting enrichment analysis, we explained the molecules potentially interacting with *AHSA1* are mainly involved in RNA transport, spliceosome and *HIF-1α* signaling pathway. The pathways above play a significant role in tumor development (36–38). This also indicated the significant involvement of *AHSA1* in the progression of tumor. The current research highlighted that the abnormal expression of *AHSA1* was closely related to the prognosis of patients with hepatocellular carcinoma. Therefore, further analysis of the association between the expression of *AHSA1* and the clinicopathological stage was carried out, and to facilitate the application of *AHSA1* in the prognosis assessment of hepatocellular carcinoma, a nomography was constructed. Previous studies have shown that *AHSA1* inhibition can significantly inhibit the proliferation and viability of breast cancer cells (39). The results of this study showed that *AHSA1* knockdown significantly reduced the proliferation activity, migration and invasion ability of Huh7 and HCCLM3 cells. Meanwhile, *AHSA1* knockdown significantly reduced the expression of *Hsp90AA1*. And *Hsp90AA1*, as the autophagy-related genes, plays key roles in the autophagy pathway and tumorigenesis. This result suggests *AHSA1* might plays a role in regulating autophagy.

*AHSA1* is abnormally upregulated in different tumor tissues, and its anomalous expression is related to the prognosis of tumors. The abnormal expression of *AHSA1* is related to the infiltration of immune cells, the expression of immune checkpoints, and the expression of various tumor pathway molecules in pan-cancer. Moreover, knockdown of *AHSA1* can affect the proliferation and transformation of hepatocellular carcinoma cells. Therefore, *AHSA1* can be used as a potential prognostic biomarker.

## Data Availability Statement

The original contributions presented in the study are included in the article/**Supplementary Material**. Further inquiries can be directed to the corresponding author.

## Ethics Statement

The studies involving human participants were reviewed and approved by Yue Bei People’s Hospital. The patients/participants provided their written informed consent to participate in this study.

## Author Contributions

JL and WL both involved in drafting the manuscript. JL and WL took part in the design and data collection process of the study and are responsible for the content of the manuscript. All authors read and approved the final manuscript.

## Conflict of Interest

The authors declare that the research was conducted in the absence of any commercial or financial relationships that could be construed as a potential conflict of interest.

## Publisher’s Note

All claims expressed in this article are solely those of the authors and do not necessarily represent those of their affiliated organizations, or those of the publisher, the editors and the reviewers. Any product that may be evaluated in this article, or claim that may be made by its manufacturer, is not guaranteed or endorsed by the publisher.

## Supplementary Material

Supplementary Figure 1 Differential expression analysis of *Hsp90AA1* in Pan-cancer. **(A)** Expression of *Hsp90AA1* mRNA in pan-cancer. **(B)** The expression differences of *Hsp90AA1* in tumor and corresponding adjacent tissues were compared with paired analysis. Mann-Whitney U test was used for this analysis, ns, p≥0.05; * p< 0.05; ** p<0.01; *** p<0.001.

Supplementary Figure 2 Real time PCR **(A)** and Weston blot **(B)** were adopted to evaluate the effects of *AHSA1* knockdown on the expression of *Hsp90AA1.* ns, p≥0.05; * p< 0.05; ** p<0.01; *** p<0.001.
