# Supplementary figures and images for: The Prognostic and Immunotherapeutic Significance of AHSA1 in Pan-Cancer, and Its Relationship With the Proliferation and Metastasis of Hepatocellular Carcinoma
Source: Front Immunol. 2022 Jun 10;13:845585. doi: 10.3389/fimmu.2022.845585 (PMC9226343; doi:10.3389/fimmu.2022.845585)

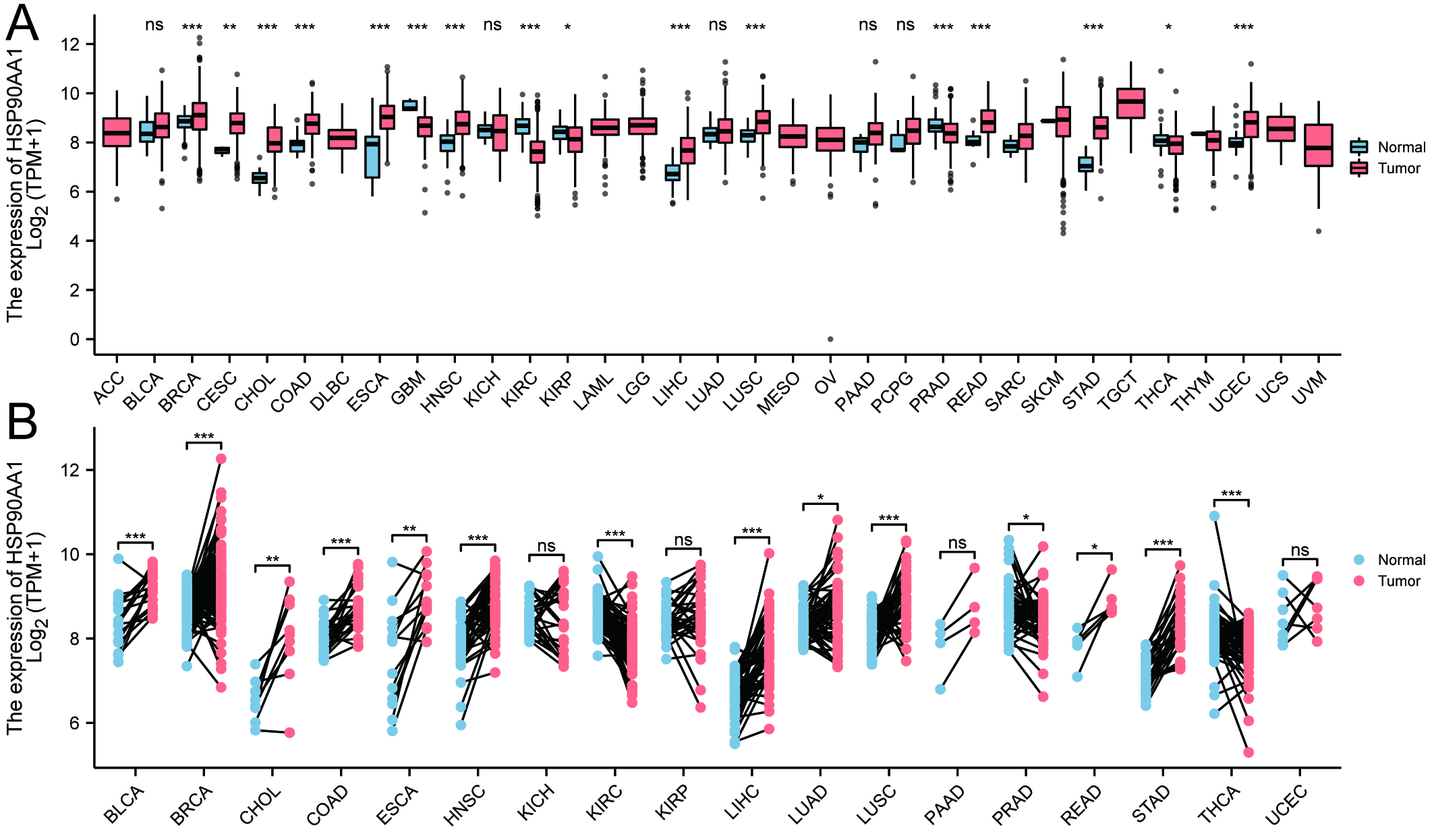

Supplement: Supplementary Figure 1 — Differential expression analysis of Hsp90AA1 in Pan-cancer. (A) Expression of Hsp90AA1 mRNA in pan-cancer. (B) The expression differences of Hsp90AA1 in tumor and corresponding adjacent tissues were compared with paired analysis. Mann-Whitney U test was used for this analysis, ns, p≥0.05; * p< 0.05; ** p<0.01; *** p<0.001. [file Image_1.tif]

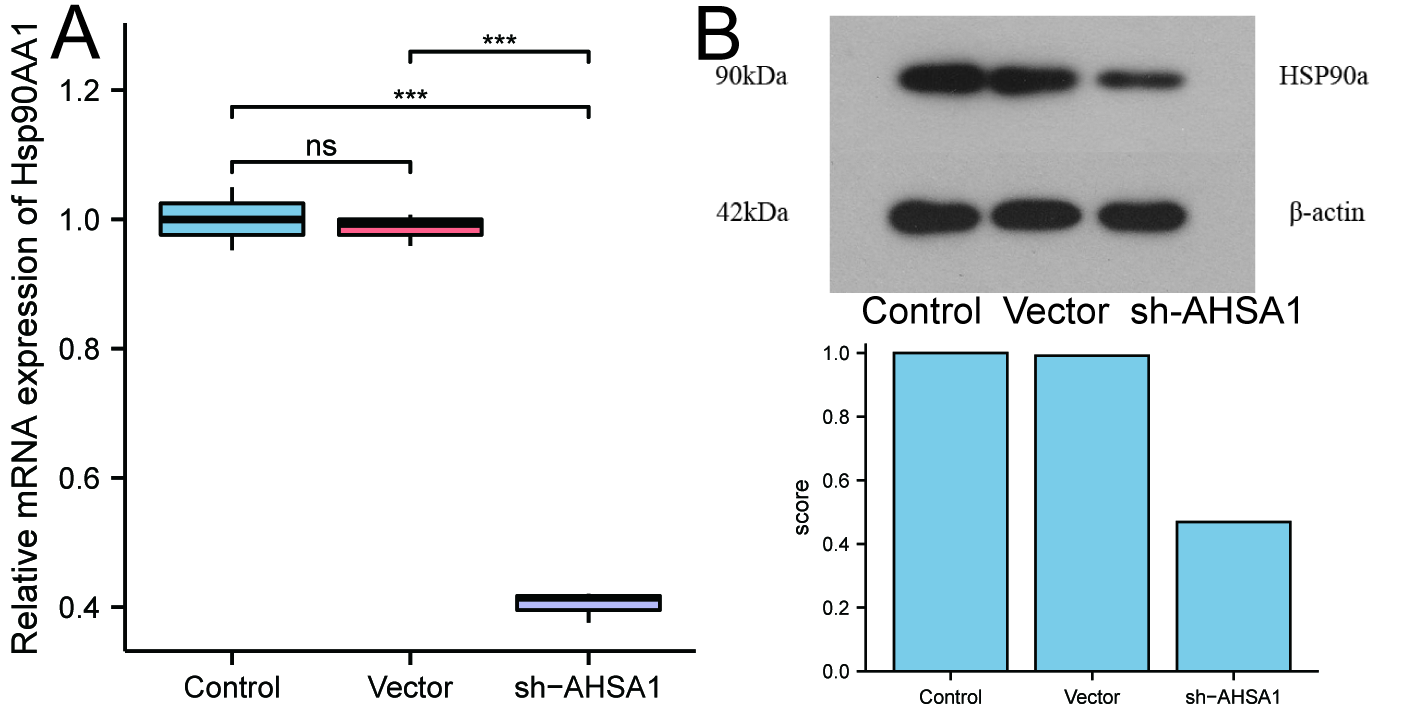

Supplement: Supplementary Figure 2 — Real time PCR (A) and Weston blot (B) were adopted to evaluate the effects of AHSA1 knockdown on the expression of Hsp90AA1. ns, p≥0.05; * p< 0.05; ** p<0.01; *** p<0.001. [file Image_2.tif]
